# Supplementary material for: Investigating Lignin-Derived Monomers and Oligomers in Low-Molecular-Weight Fractions Separated from Depolymerized Black Liquor Retentate by Membrane Filtration
Source: Molecules. 2021 May 13;26(10):2887. doi: 10.3390/molecules26102887 (PMC8152784; doi:10.3390/molecules26102887)
Supplement: Supplementary file 1 [file molecules-26-02887-s001.zip › Supplementary Materials_20210408_1.pdf]

# Supplementary Materials

# Title

Investigating lignin-derived monomers and oligomers in low-molecular-weight fractions separated from depolymerized black liquor retentate by membrane filtration

# Authors

**Kena Li [1,†], Jens Prothmann [2,†], Margareta Sandahl [2], Sara Blomberg [1], Charlotta Turner [2] and Christian Hulteberg [1,*]**

[1] Lund University, Department of Chemical Engineering, P.O. Box 124, SE-21100 Lund, Sweden; kena.li@chemeng.lth.se; sara.blomberg@chemeng.lth.se

[2] Lund University, Department of Chemistry, Centre for Analysis and Synthesis, P.O. Box 124, SE-22100 Lund, Sweden; Jens.prothmann@chem.lu.se; margareta.sandahl@chem.lu.se; charlotta.turner@chem.lu.se

* Correspondence: christian.hulteberg@chemeng.lth.se; Tel: +46 46 222 8273

† The authors contributed equally to this work.

# Contents

**Table S1.** Identified phenolic compounds in the BDM170 sample including the detected $[M-H]^-$, determined chemical formulas, classified class, obtained mass difference, obtained $^{13}C$ ratio, ring double bound (RDB) equivalent, retention times (RTs), obtained $MS^n$ spectra (*: $MS^n$ of identified fragment) [1].

**Table S2.** Identified phenolic compounds in the BDM190 sample including the detected $[M-H]^-$, determined chemical formulas, classified class, obtained mass difference, obtained $^{13}C$ ratio, ring double bound (RDB) equivalent, retention times (RTs), obtained $MS^n$ spectra (*: $MS^n$ of identified fragment).

**Table S3.** Identified phenolic compounds in the BDM210 sample including the detected $[M-H]^-$, determined chemical formulas, classified class, obtained mass difference, obtained $^{13}C$ ratio, ring double bound (RDB) equivalent, retention times (RTs), obtained $MS^n$ spectra (*: $MS^n$ of identified fragment).

**Table S4.** Identified phenolic compounds in the 2BDM190 sample including the detected $[M-H]^-$, determined chemical formulas, classified class, obtained mass difference, obtained $^{13}C$ ratio, ring double bound (RDB) equivalent, retention times (RTs), obtained $MS^n$ spectra (*: $MS^n$ of identified fragment).

**Figure S1.** BPI of BDM170 (data already presented previously in Prothmann et al. 2020 [1].

**Figure S2.** BPI of sample BDM210.

**Figure S3.** Van-Krevelen plot of sample BDM170.

**Figure S4.** Van-Krevelen plot of sample BDM190.

**Figure S5.** Van-Krevelen plot of sample BDM210.

**Figure S6.** Van-Krevelen plot of sample 2BDM190.

**Table S1.** Identified phenolic compounds in the BDM170 sample including the detected $[M-H]^-$, determined chemical formulas, classified class, obtained mass difference, obtained $^{13}C$ ratio, ring double bound (RDB) equivalent, retention times (RTs), obtained $MS^n$ spectra (*: $MS^n$ of identified fragment) [1].

| [M-H]- | Chemical formula (neutral) | Classified class | Mass diff. (mDa) | $^{13}C$ ratio | RDB | RTs (min) | $MS^n$ | Suggested compound | Identification confidence level |
|---|---|---|---|---|---|---|---|---|---|
| 121.0295 | C7H6O2 | Monomer | 0.0 | 1.10 | 5.0 | 2.51 | | 4-hydroxybenzaldehyde | 1 |
| 135.0451 | C8H8O2 | Monomer | 0.0 | 1.04 | 5.0 | 2.54 | $MS^2$ | 4-hydroxyacetophenone | 1 |
| 137.0243 | C7H6O3 | Monomer | -0.1 | 0.96 | 5.0 | 4.05 | $MS^2$ | 4-hydroxybenzoic acid | 1 |
| 151.0399 | C8H8O3 | Monomer | -0.1 | 1.00 | 5.0 | 1.25 | $MS^3$ | Vanillin | 1 |
| 165.0554 | C9H10O3 | Monomer | -0.3 | 0.97 | 5.0 | 1.25 | | Acetovanillone | 1 |
| | | | | 1.00 | | 2.23 | $MS^2$ | Homovanillin | 2 |
| 167.0347 | C8H8O4 | Monomer | -0.3 | 1.03 | 5.0 | 1.62 | | 3,4-dihydroxy-5-methoxybenzaldehyde | 3 |
| | | | | 1.04 | | 4.08 | | 2-(3,4-dihydroxy acetaldehyde | 3 |
| 175.0761 | C11H12O2 | Monomer | -0.3 | 0.97 | 6.0 | 2.85 | | (5-ethylbenzo-furan-3-yl) methanol | 3 |
| 181.0502 | C9H10O4 | Monomer | -0.4 | 1.06 | 5.0 | 1.63 | $MS^3$ | Syringaldehyde | 1 |
| | | | | 0.95 | | 3.68 | $MS^3$ | 3,4-dihydroxy-5-methoxy-acetophenone | 2 |
| 195.0658 | C10H12O4 | Monomer | -0.5 | 1.00 | 5.0 | 1.63 | | Acetosyringone | 1 |
| | | | | 1.00 | | 3.22 | | 1-(3,4-dihydroxy-5-methoxyphenyl) propan-2-one | 3 |
| 203.0709 | C12H12O3 | Monomer | -0.4 | 1.00 | 7.0 | 2.57 | | (7-methoxy-5-vinylbenzofuran -3-yl) methanol | 3 |
| 233.0270 | C12H10O3S | Dimer | -0.8 | 1.03 | | 2.14 | | | 3 |
| 243.0653 | C14H12O4 | Dimer | -1.0 | 1.08 | 9.0 | 3.40 | | | 3 |
| | | | | 0.93 | | 3.52 | | | 3 |
| 245.0810 | C14H14O4 | Dimer | -0.9 | 0.88 | 8.0 | 5.26 | | | 3 |

| | | | | | | | | | |
|---|---|---|---|---|---|---|---|---|---|
| 257.0447 | C14H10O5 | Dimer | -0.8 | 1.00 | 10.0 | 3.27 | | | 3 |
| 257.0812 | C15H14O4 | Dimer | -0.7 | 0.95 | 9.0 | 2.85 | | | 3 |
| 259.0604 | C14H12O5 | Dimer | -0.8 | 0.88 | 9.0 | 3.62 | | | 3 |
| 271.0604 | C15H12O5 | Dimer | -0.8 | 1.00 | 10.0 | 3.13 | | | 3 |
| | | | | 1.04 | | 4.53 | $MS^3$ | | 3 |
| 273.0759 | C15H14O5 | Dimer | -0.9 | 1.00 | 9.0 | 3.28 | $MS^3$ | | 3 |
| 273.1124 | C16H18O4 | Dimer | -0.8 | ° | 8.0 | 3.99 | | | 3 |
| | | | | 0.92 | | 4.19 | | | 3 |
| 287.0915 | C16H16O5 | Dimer | -1.0 | 0.92 | 9.0 | 2.94 | $MS^3$ | | 2 |
| | | | | 0.94 | | 3.13 | | | 3 |
| 299.0916 | C17H16O5 | Dimer | -0.9 | 0.86 | 10.0 | 3.50 | | | 3 |
| 301.0707 | C16H14O6 | Dimer | -1.0 | 0.96 | 10.0 | 1.26 | | | 3 |
| | | | | 1.03 | | 3.93 | $MS^3$ | | 2 |
| | | | | 1.00 | | 4.25 | $MS^3$ | | 3 |
| 301.1071 | C17H18O5 | Dimer | -1.0 | 0.99 | 9.0 | 2.99 | $MS^3$ | | 2 |
| | | | | 0.92 | | 5.48 | | | 3 |
| 301.1436 | C18H22O4 | Dimer | -0.9 | 1.00 | 8.0 | 4.87 | | | 3 |
| 303.0865 | C16H16O6 | Dimer | -0.9 | 0.90 | 9.0 | 3.69 | | | 3 |
| 313.1434 | C19H22O4 | Dimer | -1.1 | 0.98 | 9.0 | 5.18 | $MS^3$ | | 3 |
| | | | | 1.00 | | 5.75 | $MS^3$ | | 3 |
| 315.0862 | C17H16O6 | Dimer | -1.2 | 1.07 | 10.0 | 1.26 | | | 3 |
| | | | | 0.89 | | 2.78 | | | 3 |
| | | | | 1.08 | | 4.00 | $MS^3$ | | 3 |
| | | | | 0.85 | | 5.12 | | | 3 |
| 315.1227 | C18H20O5 | Dimer | -1.1 | 1.05 | 9.0 | 3.13 | $MS^3$ | | 2 |
| 317.1021 | C17H18O6 | Dimer | -0.9 | 0.85 | 9.0 | 3.60 | | | 3 |
| 323.0915 | C19H16O5 | Dimer | -1.0 | 0.91 | 12.0 | 3.86 | | | 3 |

| | | | | | | | | | |
|---|---|---|---|---|---|---|---|---|---|
| 329.1018 | C18H18O6 | Dimer | -1.2 | 1.00 | 10.0 | 1.26 | | | 3 |
| | | | | 0.98 | | 3.80 | $MS^2$ | | 2 |
| | | | | 1.00 | | 3.87 | $MS^2$ | | 3 |
| | | | | 0.99 | | 4.16 | $MS^2$ | | 3 |
| | | | | 0.98 | | 4.99 | | | 3 |
| 329.1383 | C19H22O5 | Dimer | -1.1 | 0.99 | 9.0 | 2.99 | | | 3 |
| | | | | 1.06 | | 6.18 | $MS^3$ | | 3 |
| 331.1176 | C18H20O6 | Dimer | -1.1 | 0.95 | 9.0 | 3.37 | | | 3 |
| | | | | 1.00 | | 4.69 | $MS^3$ | | 2 |
| 343.1176 | C19H20O6 | Dimer | -1.1 | 1.05 | 10.0 | 3.72 | | | 3 |
| 345.1332 | C19H22O6 | Dimer | -1.0 | 0.89 | 9.0 | 3.52 | | | 3 |
| | | | | 1.05 | | 6.86 | $MS^3$ | | 3 |
| 347.1124 | C18H20O7 | Dimer | -1.2 | 0.95 | 9.0 | 4.09 | | | 3 |
| | | | | 1.07 | | 5.29 | $MS^3$ | | 3 |
| 351.1228 | C21H20O5 | Dimer | -1.0 | 1.13 | 12.0 | 4.27 | | | 3 |
| 355.1175 | C20H20O6 | Dimer | -1.2 | 0.91 | 11.0 | 4.12 | | | 3 |
| | | | | 0.90 | | 4.63 | | | 3 |
| 359.1488 | C20H24O6 | Dimer | -1.2 | 1.00 | 9.0 | 4.47 | $MS^3$ | | 2 |
| | | | | 0.96 | | 4.66 | | | 3 |
| | | | | 0.97 | | 5.44 | | | 3 |
| 365.1018 | C21H18O6 | Dimer | -1.2 | 0.92 | 13.0 | 4.84 | | | 3 |
| | | | | 1.00 | | 5.48 | | | 3 |
| 373.1281 | C20H22O7 | Dimer | -1.2 | 1.09 | 10.0 | 3.66 | | | 3 |
| | | | | 0.94 | | 4.46 | | | 3 |
| | | | | 0.93 | | 5.29 | | | 3 |
| | | | | 0.88 | | 5.45 | | | 3 |
| | | | | 1.00 | | 5.50 | | | 3 |

| | | | | | | | | | |
|---|---|---|---|---|---|---|---|---|---|
| 379.1386 | C19H24O8 | Dimer | -1.2 | 0.94 | 8.0 | 5.22 | | | 3 |
| 399.1437 | C22H24O7 | Dimer | -1.2 | 0.92 | 11.0 | 4.05 | | | 3 |
| 419.1700 | C22H28O8 | Dimer | -1.1 | 0.88 | 9.0 | 4.77 | | | 3 |
| | | | | 0.89 | | 4.83 | | | 3 |
| | | | | 0.96 | | 5.34 | | | 3 |

**Table S2.** Identified phenolic compounds in the BDM190 sample including the detected [M-H]⁻, determined chemical formulas, classified class, obtained mass difference, obtained $^{13}C$ ratio, ring double bound (RDB) equivalent, retention times (RTs), obtained $MS^n$ spectra (*: $MS^n$ of identified fragment).

| [M-H]- | Chemical formula (neutral) | Classified class | Mass diff. (mDa) | $^{13}C$ ratio | RDB | RTs (min) | $MS^n$ | Suggested compound | Identification confidence level |
|---|---|---|---|---|---|---|---|---|---|
| 109.0296 | C6H6O2 | Monomer | 0.1 | 0.96 | 4.0 | 0.56 | | Catechol | 3 |
| 121.0295 | C7H6O2 | Monomer | 0.0 | 1.00 | 5.0 | 2.52 | | 4-hydroxybenzaldehyde | 1 |
| 135.0450 | C8H8O2 | Monomer | 0.0 | 1.08 | 5.0 | 2.55 | | 4-hydroxyacetophenone | 1 |
| 137.0243 | C7H6O3 | Monomer | -0.1 | 0.86 | 5.0 | 1.24 | | | 3 |
| | | | | 1.00 | | 4.02 | | | 3 |
| 151.0399 | C8H8O3 | Monomer | -0.1 | 1.00 | 5.0 | 1.25 | $MS^3$ | Vanillin | 1 |
| | | | | 0.97 | | 3.62 | | | 3 |
| 161.0605 | C10H10O2 | Monomer | -0.3 | 0.89 | 6.0 | 2.83 | $MS^2$ | | 2 |
| | | | | 0.91 | | 2.90 | | | 3 |
| | | | | 0.94 | | 2.95 | | | 3 |
| | | | | 1.08 | | 3.08 | | | 3 |
| 165.0555 | C9H10O3 | Monomer | -0.2 | 1.03 | 5.0 | 1.25 | | Acetovanillone | 1 |
| | | | | 1.09 | | 2.24 | $MS^3$ | | 2 |
| 167.0346 | C8H8O4 | Monomer | -0.4 | 0.92 | 5.0 | 1.24 | | | 3 |
| | | | | 1.00 | | 1.63 | | | 3 |
| 175.0761 | C11H12O2 | Monomer | -0.3 | 0.91 | 6.0 | 2.69 | | | 3 |
| | | | | 0.89 | | 2.80 | | | 3 |
| | | | | 1.08 | | 2.86 | | | 3 |
| 177.0554 | C10H10O3 | Monomer | -0.3 | 0.92 | 6.0 | 3.55 | | | 3 |
| | | | | 0.92 | | 3.65 | | | 3 |
| 179.0709 | C10H12O3 | Monomer | -0.4 | 0.93 | 5.0 | 1.20 | | | 3 |
| | C10H12O3 | | | 0.92 | | 2.19 | | | 3 |

| | | | | | | | | | |
|---|---|---|---|---|---|---|---|---|---|
| | C10H12O3 | | | 0.91 | | 2.81 | | | 3 |
| | C10H12O3 | | | 0.85 | | 3.74 | | | 3 |
| 181.0503 | C9H10O4 | Monomer | -0.3 | 1.00 | 5.0 | 1.63 | $MS^3$ | Syringaldehyde | 1 |
| 183.0295 | C8H8O5 | Monomer | -0.4 | 0.95 | 5.0 | 1.24 | | | 3 |
| 191.0709 | C11H12O3 | Monomer | -0.4 | 0.89 | 6.0 | 1.61 | | | 3 |
| | | | | 0.92 | | 1.77 | | | 3 |
| | | | | 0.96 | | 3.62 | | | 3 |
| 195.0658 | C10H12O4 | Monomer | -0.5 | 1.01 | 5.0 | 1.63 | | Acetosyringone | 1 |
| | | | | 1.02 | | 2.93 | | | 3 |
| | | | | 0.85 | | 3.21 | | | 3 |
| 213.0551 | C13H10O3 | Monomer | -0.6 | 0.97 | 9.0 | 3.63 | | | 3 |
| | | | | 0.86 | | 3.56 | | | 3 |
| 241.0499 | C14H10O4 | Dimer | -0.7 | 0.92 | 10.0 | 3.30 | | | 3 |
| | | | | 1.00 | | 4.70 | | | 3 |
| 243.0654 | C14H12O4 | Dimer | -0.9 | 1.09 | 9.0 | 3.40 | | | 3 |
| | | | | 1.00 | | 3.52 | | | 3 |
| 245.0810 | C14H14O4 | Dimer | -0.7 | 0.86 | 8.0 | 0.56 | | | 3 |
| | | | | 0.97 | | 2.53 | | | 3 |
| 255.0656 | C15H12O4 | Dimer | -0.7 | 0.85 | 10.0 | 3.05 | | | 3 |
| 257.0812 | C15H14O4 | Dimer | -0.7 | 0.95 | 9.0 | 2.87 | | | 3 |
| | | | | 0.85 | | 3.28 | | | 3 |
| | | | | 0.87 | | 3.34 | | | 3 |
| | | | | 0.90 | | 3.62 | | | 3 |
| | | | | 0.85 | | 3.80 | | | 3 |
| | | | | 0.90 | | 4.10 | | | 3 |
| 259.0604 | C14H12O5 | Dimer | -0.8 | 0.95 | 9.0 | 3.50 | | | 3 |
| | | | | 1.04 | | 3.62 | | | 3 |

| | | | | | | | | | |
|---|---|---|---|---|---|---|---|---|---|
| 259.0969 | C15H16O4 | Dimer | -0.7 | 0.86 | 8.0 | 0.59 | | | 3 |
| | | | | 0.96 | | 2.18 | | | 3 |
| | | | | 0.86 | | 4.67 | | | 3 |
| 271.0604 | C15H12O5 | Dimer | -0.8 | 0.89 | 10.0 | 4.54 | | | 3 |
| 271.0967 | C16H16O4 | Dimer | -0.9 | 0.88 | 9.0 | 3.74 | | | 3 |
| | | | | 0.90 | | 4.04 | | | 3 |
| 273.0759 | C15H14O5 | Dimer | -0.9 | 1.00 | 9.0 | 3.28 | $MS^3$ | | 3 |
| | | | | 0.97 | | 3.58 | $MS^3$ | | 3 |
| | | | | 1.00 | | 4.25 | | | 3 |
| 273.1123 | C16H18O4 | Dimer | -0.9 | 0.87 | 8.0 | 0.59 | | | 3 |
| | | | | 0.89 | | 2.06 | | | 3 |
| | | | | 0.93 | | 4.19 | | | 3 |
| 275.0915 | C15H16O5 | Dimer | -1.0 | 0.89 | 8.0 | 3.78 | | | 3 |
| | | | | 0.85 | | 5.29 | | | 3 |
| 285.0760 | C16H14O5 | Dimer | -0.8 | 0.96 | 10.0 | 4.22 | | | 3 |
| 285.1124 | C17H18O4 | Dimer | -0.8 | 0.86 | 9.0 | 4.03 | | | 3 |
| 287.0915 | C16H16O5 | Dimer | -1.0 | 0.88 | 9.0 | 2.95 | | | 3 |
| | | | | 1.00 | | 3.15 | | | 3 |
| 287.1280 | C17H20O4 | Dimer | -0.9 | 0.90 | 8.0 | 4.15 | | | 3 |
| 289.0708 | C15H14O6 | Dimer | -0.9 | 0.99 | 9.0 | 3.66 | | | 3 |
| 299.0916 | C17H16O5 | Dimer | -0.9 | 1.00 | 10.0 | 4.06 | | | 3 |
| 301.0707 | C16H14O6 | Dimer | -1.0 | 1.00 | 10.0 | 1.25 | | | 3 |
| | | | | 1.00 | | 3.94 | $MS^3$ | | 2 |
| | | | | 0.96 | | 4.04 | | | 3 |
| | | | | 1.01 | | 4.26 | $MS^3$ | | 3 |
| 301.1071 | C17H18O5 | Dimer | -1.0 | 1.01 | 9.0 | 3.02 | | | 3 |
| | | | | 0.97 | | 3.08 | | | 3 |

| | | | | | | | | | |
|---|---|---|---|---|---|---|---|---|---|
| 301.1435 | C18H22O4 | Dimer | -1.0 | 0.93 | 8.0 | 4.87 | | | 3 |
| 303.0864 | C16H16O6 | Dimer | -1.0 | 0.93 | 9.0 | 3.69 | $MS^3$ | | 3 |
| 315.0863 | C17H16O6 | Dimer | -1.1 | 1.06 | 10.0 | 1.25 | | | 3 |
| | | | | 1.00 | | 2.78 | | | 3 |
| | | | | 0.93 | | 3.77 | | | 3 |
| | | | | 1.00 | | 4.00 | $MS^3$ | | 3 |
| 315.1227 | C18H20O5 | Dimer | -1.1 | 0.94 | 9.0 | 2.86 | | | 3 |
| | | | | 0.97 | | 2.93 | | | 3 |
| | | | | 1.00 | | 3.14 | | | 3 |
| | | | | 0.93 | | 3.44 | | | 3 |
| 317.1021 | C17H18O6 | Dimer | -0.9 | 1.00 | 9.0 | 3.59 | | | 3 |
| | | | | 0.95 | | 4.88 | | | 3 |
| 325.1071 | C19H18O5 | Dimer | -1.0 | 0.93 | 11.0 | 4.03 | | | 3 |
| 329.1018 | C18H18O6 | Dimer | -1.1 | 1.00 | 10.0 | 1.25 | | | 3 |
| | | | | 1.01 | | 3.80 | $MS^3$ | | 2 |
| | | | | 0.96 | | 3.87 | $MS^3$ | | 3 |
| 329.1382 | C19H22O5 | Dimer | -1.1 | 0.94 | 9.0 | 2.99 | | | 3 |
| | | | | 1.04 | | 6.17 | | | 3 |
| 331.1176 | C18H20O6 | Dimer | -1.1 | 0.96 | 9.0 | 3.39 | | | 3 |
| | | | | 1.00 | | 3.50 | | | 3 |
| | | | | 0.87 | | 4.43 | | | 3 |
| | | | | 0.97 | | 4.50 | | | 3 |
| | | | | 0.92 | | 4.59 | | | 3 |
| | | | | 0.96 | | 4.69 | $MS^3$ | | 2 |
| 335.1125 | C17H20O7 | Dimer | -1.1 | 1.07 | 8.0 | 4.98 | | | 3 |
| | | | | 1.05 | | 5.03 | | | 3 |
| | | | | 0.87 | | 5.22 | | | 3 |

| | | | | | | | | | |
|---|---|---|---|---|---|---|---|---|---|
| | | | | 1.15 | | 5.26 | | | 3 |
| 339.1227 | C20H20O5 | Dimer | -1.1 | 0.90 | 11.0 | 3.78 | | | 3 |
| 343.1175 | C19H20O6 | Dimer | -1.2 | 0.91 | 10.0 | 2.77 | | | 3 |
| | | | | 1.00 | | 3.72 | | | 3 |
| | | | | 1.01 | | 4.84 | | | 3 |
| | | | | 1.00 | | 4.89 | | | 3 |
| 345.0968 | C18H18O7 | Dimer | -1.2 | 1.00 | 10.0 | 3.78 | | | 3 |
| 345.1332 | C19H22O6 | Dimer | -1.1 | 1.00 | 9.0 | 3.52 | | | 3 |
| | | | | 1.07 | | 4.62 | $MS^3$ | | 2 |
| 347.1124 | C18H20O7 | Dimer | -1.2 | 0.91 | 9.0 | 4.02 | | | 3 |
| | | | | 1.07 | | 4.10 | $MS^3$ | | 2 |
| | | | | 1.05 | | 5.30 | | | 3 |
| 351.1225 | C21H20O5 | Dimer | -1.3 | 0.95 | 12.0 | 4.26 | | | 3 |
| 355.1175 | C20H20O6 | Dimer | -1.2 | 0.91 | 11.0 | 4.10 | | | 3 |
| | | | | 0.90 | | 4.63 | | | 3 |
| 359.1125 | C19H20O7 | Dimer | -1.1 | 0.87 | 10.0 | 6.89 | | | 3 |
| 359.1488 | C20H24O6 | Dimer | -1.2 | 0.93 | 9.0 | 4.47 | | | 3 |
| 365.1019 | C21H18O6 | Dimer | -1.1 | 1.00 | 13.0 | 4.84 | | | 3 |
| | | | | 0.92 | | 5.48 | | | 3 |
| 373.1283 | C20H22O7 | Dimer | -1.0 | 1.00 | 10.0 | 5.49 | | | 3 |
| 375.1437 | C20H24O7 | Dimer | -1.2 | 1.00 | 9.0 | 3.37 | | | 3 |
| | | | | 0.95 | | 3.63 | | | 3 |
| | | | | 1.00 | | 4.66 | | | 3 |
| | | | | 1.00 | | 4.69 | | | 3 |
| 377.1230 | C19H22O8 | Dimer | -1.2 | 1.01 | 9.0 | 4.16 | | | 3 |
| 383.1488 | C22H24O6 | Dimer | -1.2 | 0.89 | 11.0 | 4.66 | | | 3 |
| 403.1386 | C21H24O8 | Dimer | -1.2 | 0.95 | 10.0 | 3.93 | | | 3 |

| | | | | | | | | | |
|---|---|---|---|---|---|---|---|---|---|
| | | | | 0.93 | 10.0 | 4.22 | | | 3 |
| 419.1702 | C22H28O8 | Dimer | -0.9 | 0.92 | 9.0 | 4.62 | | | 3 |
| | | | | 1.00 | | 4.84 | | | 3 |
| | | | | 0.98 | | 5.33 | | | 3 |
| 429.1543 | C23H26O8 | Dimer | -1.2 | 0.88 | 11.0 | 4.79 | | | 3 |

**Table S3.** Identified phenolic compounds in the BDM210 sample including the detected [M-H]-, determined chemical formulas, classified class, obtained mass difference, obtained $^{13}C$ ratio, ring double bound (RDB) equivalent, retention times (RTs), obtained $MS^n$ spectra (*: $MS^n$ of identified fragment).

| [M-H]- | Chemical formula (neutral) | Classified class | Mass diff. (mDa) | $^{13}C$ ratio | RDB | RTs (min) | $MS^n$ | Suggested compound | Identification confidence level |
|---|---|---|---|---|---|---|---|---|---|
| 121.0295 | $C_7H_6O_2$ | Monomer | 0.0 | 1.15 | 5.0 | 2.52 | | 4-hydroxybenzaldehyde | 1 |
| 135.0451 | $C_8H_8O_2$ | Monomer | 0.0 | 1.13 | 5.0 | 2.12 | | | 3 |
| | | | | 1.09 | | 2.44 | | | 3 |
| | | | | 1.00 | | 2.55 | | 4-hydroxyacetophenone | 1 |
| 137.0243 | $C_7H_6O_3$ | Monomer | -0.1 | 0.88 | 5.0 | 1.25 | | | 3 |
| | | | | 0.86 | | 1.56 | | | 3 |
| | | | | 0.88 | | 1.63 | | | 3 |
| | | | | 0.98 | | 4.01 | | 4-hydroxybenzoic acid | 1 |
| 145.0294 | $C_9H_6O_2$ | Monomer | -0.1 | 0.87 | 7.0 | 2.83 | | | 3 |
| 147.0450 | $C_9H_8O_2$ | Monomer | -0.1 | 1.00 | 6.0 | 3.02 | | | 3 |
| 149.0606 | $C_9H_{10}O_2$ | Monomer | -0.2 | 0.93 | 5.0 | 1.91 | | | 3 |
| | | | | 0.91 | | 2.49 | $MS^2$ | | 2 |
| 151.0398 | $C_8H_8O_3$ | Monomer | -0.2 | 1.00 | 5.0 | 1.26 | $MS^3$ | Vanillin | 1 |
| 161.0605 | $C_{10}H_{10}O_2$ | Monomer | -0.3 | 1.00 | 6.0 | 2.69 | | | 3 |
| | | | | 1.03 | | 2.83 | $MS^2$ | | 2 |
| | | | | 1.03 | | 2.90 | | | 3 |
| | | | | 1.09 | | 2.94 | | | 3 |
| | | | | 1.00 | | 3.06 | | | 3 |
| 165.0554 | $C_9H_{10}O_3$ | Monomer | -0.3 | 1.07 | 5.0 | 1.25 | | Acetovanillone | 1 |
| | | | | 1.03 | | 2.24 | $MS^3$ | | 2 |
| 175.0761 | $C_{11}H_{12}O_2$ | Monomer | -0.3 | 1.07 | 6.0 | 2.60 | | | 3 |
| | | | | 0.94 | | 2.69 | | | 3 |

| | | | | | | | | | |
|---|---|---|---|---|---|---|---|---|---|
| | | | | 1.00 | | 2.81 | | | 3 |
| | | | | 1.05 | | 2.85 | $MS^2$ | | 2 |
| | | | | 0.85 | | 2.96 | | | 3 |
| | | | | 1.15 | | 3.03 | | | 3 |
| 177.0554 | C10H10O3 | Monomer | -0.3 | 0.98 | 6.0 | 1.78 | | Coniferyl aldehyde | 1 |
| | | | | 0.96 | | 2.74 | | | 3 |
| | | | | 0.97 | | 3.55 | | | 3 |
| | | | | 1.10 | | 3.66 | $MS^2$ | | 2 |
| 179.0347 | C9H8O4 | Monomer | -0.3 | 1.02 | 6.0 | 3.45 | | | 3 |
| 179.0710 | C10H12O3 | Monomer | -0.3 | 1.06 | 5.0 | 1.20 | | | 3 |
| | | | | 0.93 | | 2.18 | | | 3 |
| | | | | 0.90 | | 2.81 | | | 3 |
| | | | | 0.88 | | 3.03 | | Coniferyl alcohol | 1 |
| | | | | 0.88 | | 3.45 | | | 3 |
| | | | | 0.86 | | 4.01 | | | 3 |
| 181.0501 | C9H10O4 | Monomer | -0.5 | 0.91 | 5.0 | 1.25 | | | 3 |
| | | | | 1.00 | | 1.63 | $MS^3$ | Syringaldehyde | 1 |
| 183.0294 | C8H8O5 | Monomer | -0.5 | 1.02 | 5.0 | 1.25 | | | 3 |
| 185.0603 | C12H10O2 | Monomer | -0.5 | 1.15 | 8.0 | 1.56 | | | 3 |
| | | | | 1.08 | | 2.69 | $MS^2$ | | 2 |
| | | | | 1.07 | | 3.86 | | | 3 |
| 187.0396 | C11H8O3 | Monomer | -0.4 | 1.00 | 8.0 | 3.41 | | | 3 |
| 187.0760 | C12H12O2 | Monomer | -0.4 | 0.94 | 7.0 | 3.19 | | | 3 |
| | | | | 0.92 | | 3.23 | | | 3 |
| 191.0709 | C11H12O3 | Monomer | -0.4 | 0.93 | 6.0 | 3.38 | | | 3 |
| | | | | 1.05 | | 3.51 | | | 3 |
| | | | | 1.02 | | 3.61 | $MS^3$ | | 2 |

| | | | | | | | | | |
|---|---|---|---|---|---|---|---|---|---|
| 195.0446 | C13H8O2 | Monomer | -0.5 | 0.91 | 10.0 | 4.30 | | | 3 |
| 195.0657 | C10H12O4 | Monomer | -0.6 | 0.99 | 5.0 | 1.63 | | Acetosyringone | 1 |
| | | | | 0.93 | | 2.87 | | | 3 |
| | | | | 0.94 | | 2.93 | | | 3 |
| | | | | 1.00 | | 3.00 | | | 3 |
| | | | | 1.00 | | 3.21 | | | 3 |
| 201.0552 | C12H10O3 | Monomer | -0.5 | 0.85 | 8.0 | 3.18 | | | 3 |
| 203.0709 | C12H12O3 | Monomer | -0.4 | 0.96 | 7.0 | 2.58 | | | 3 |
| | | | | 1.00 | | 3.41 | | | 3 |
| 213.0551 | C13H10O3 | Monomer | -0.6 | 1.05 | 9.0 | 3.63 | $MS^2$ | | 2 |
| 227.0343 | C13H8O4 | Monomer | -0.7 | 1.11 | 10.0 | 4.28 | | | 3 |
| 227.0706 | C14H12O3 | Dimer | -0.7 | 0.93 | 9.0 | 3.45 | | | 3 |
| | | | | 1.00 | | 3.55 | | | 3 |
| 229.0499 | C13H10O4 | Monomer | -0.7 | 1.00 | 9.0 | 5.46 | | | 3 |
| 231.0655 | C13H12O4 | Monomer | -0.8 | 0.89 | 8.0 | 0.58 | | | 3 |
| | | | | 0.90 | | 2.72 | | | 3 |
| 241.0498 | C14H10O4 | Dimer | -0.8 | 0.93 | 10.0 | 3.28 | | | 3 |
| | | | -0.8 | 1.07 | 10.0 | 4.25 | | | 3 |
| | | | -0.8 | 0.89 | 10.0 | 4.72 | | | 3 |
| 241.0865 | C15H14O3 | Dimer | -0.5 | 1.14 | 9.0 | 3.41 | | | 3 |
| 243.0654 | C14H12O4 | Dimer | -0.8 | 0.98 | 9.0 | 3.41 | | | 3 |
| | | | | 1.11 | | 3.51 | | | 3 |
| | | | | 1.00 | | 3.92 | | | 3 |
| | | | | 0.87 | | 5.29 | | | 3 |
| 243.1018 | C15H16O3 | Dimer | -0.8 | 0.90 | 8.0 | 4.14 | | | 3 |
| | | | | 0.86 | | 4.34 | | | 3 |
| 245.0810 | C14H14O4 | Dimer | -0.9 | 0.95 | 8.0 | 0.56 | | | 3 |

| | | | | | | | | | |
|---|---|---|---|---|---|---|---|---|---|
| | | | | 0.90 | | 2.07 | | | 3 |
| | | | | 1.07 | | 2.53 | | | 3 |
| | | | | 0.92 | | 3.03 | | | 3 |
| | | | | 1.00 | | 5.26 | | | 3 |
| | | | | 0.88 | | 6.20 | | | 3 |
| 255.0654 | C15H12O4 | Dimer | -0.9 | 0.89 | 10.0 | 3.05 | | | 3 |
| | | | | 0.90 | | 3.15 | | | 3 |
| | | | | 0.86 | | 4.14 | | | 3 |
| | | | | 0.97 | | 4.46 | | | 3 |
| 257.0448 | C14H10O5 | Dimer | -0.7 | 0.87 | 10.0 | 3.41 | | | 3 |
| | | | | 1.15 | | 4.27 | | | 3 |
| 257.0810 | C15H14O4 | Dimer | -0.9 | 0.97 | 9.0 | 2.87 | | | 3 |
| | | | | 0.96 | | 3.29 | | | 3 |
| | | | | 0.92 | | 3.34 | | | 3 |
| | | | | 1.05 | | 3.64 | | | 3 |
| | | | | 0.96 | | 3.79 | | | 3 |
| | | | | 0.85 | | 3.83 | | | 3 |
| 259.0603 | C14H12O5 | Dimer | -0.9 | 0.97 | 9.0 | 3.51 | | | 3 |
| | | | | 1.07 | | 3.57 | $MS^3$ | | 2 |
| 259.0966 | C15H16O4 | Dimer | -1.0 | 0.91 | 8.0 | 0.58 | | | 3 |
| 269.0812 | C16H14O4 | Dimer | -0.7 | 0.93 | 10.0 | 4.06 | | | 3 |
| | | | | 0.98 | | 4.60 | | | 3 |
| 271.0604 | C15H12O5 | Dimer | -0.8 | 1.00 | 10.0 | 4.37 | | | 3 |
| | | | | 1.02 | | 4.54 | | | 3 |
| | | | | 0.98 | | 5.44 | | | 3 |
| 271.0966 | C16H16O4 | Dimer | -1.0 | 1.00 | 9.0 | 2.87 | | | 3 |
| | | | | 0.86 | | 3.64 | | | 3 |

| | | | | | | | | | |
|---|---|---|---|---|---|---|---|---|---|
| | | | | 0.92 | | 3.73 | | | 3 |
| | | | | 0.95 | | 4.04 | | | 3 |
| | | | | 1.07 | | 4.33 | | | 3 |
| 273.0758 | C15H14O5 | Dimer | -1.0 | 0.89 | 9.0 | 2.49 | | | 3 |
| | | | | 0.92 | | 3.00 | | | 3 |
| | | | | 1.02 | | 3.29 | $MS^3$ | | 3 |
| | | | | 1.00 | | 3.47 | | | 3 |
| | | | | 1.00 | | 3.53 | | | 3 |
| | | | | 1.10 | | 3.57 | $MS^3$ | | 2 |
| | | | | 0.90 | | 4.01 | | | 3 |
| | | | | 1.02 | | 4.25 | | | 3 |
| 273.1124 | C16H18O4 | Dimer | -0.8 | 0.88 | 8.0 | 0.59 | | | 3 |
| | | | | 1.03 | | 2.40 | | | 3 |
| 275.0915 | C15H16O5 | Dimer | -1.0 | 0.93 | 8.0 | 2.84 | | | 3 |
| | | | | 0.88 | | 3.77 | | | 3 |
| 281.0811 | C17H14O4 | Dimer | -0.8 | 0.91 | 11.0 | 4.51 | | | 3 |
| 283.0967 | C17H16O4 | Dimer | -0.9 | 0.93 | 10.0 | 4.05 | | | 3 |
| | | | | 0.87 | | 4.17 | | | 3 |
| 285.0760 | C16H14O5 | Dimer | -0.8 | 0.93 | 10.0 | 4.22 | | | 3 |
| 287.0553 | C15H12O6 | Dimer | -0.8 | 1.00 | 10.0 | 4.17 | | | 3 |
| 287.0914 | C16H16O5 | Dimer | -1.1 | 0.97 | 9.0 | 2.96 | | | 3 |
| | | | | 1.00 | | 3.15 | $MS^3$ | | 3 |
| 287.1280 | C17H20O4 | Dimer | -0.9 | 0.94 | 8.0 | 0.59 | | | 3 |
| | | | | 0.86 | | 2.37 | | | 3 |
| 289.0708 | C15H14O6 | Dimer | -0.9 | 0.98 | 9.0 | 3.66 | | | 3 |
| | | | | 0.94 | | 3.73 | | | 3 |
| | | | | 1.11 | | 3.77 | | | 3 |

| | | | | | | | | | |
|---|---|---|---|---|---|---|---|---|---|
| 289.1071 | C16H18O5 | Dimer | -1.0 | 0.95 | 8.0 | 4.21 | $MS^3$ | | 3 |
| 299.0915 | C17H16O5 | Dimer | -0.9 | 1.00 | 10.0 | 4.06 | | | 3 |
| 301.0707 | C16H14O6 | Dimer | -1.0 | 1.06 | 10.0 | 1.28 | | | 3 |
| | | | | 0.92 | | 3.93 | $MS^3$ | | 2 |
| | | | | 1.02 | | 4.06 | | | 3 |
| | | | | 1.06 | | 4.25 | $MS^3$ | | 3 |
| 301.1070 | C17H18O5 | Dimer | -1.1 | 0.95 | 9.0 | 3.07 | | | 3 |
| 301.1436 | C18H22O4 | Dimer | -0.9 | 1.00 | 8.0 | 4.76 | | | 3 |
| | | | | 0.90 | | 4.86 | | | 3 |
| 303.0863 | C16H16O6 | Dimer | -1.1 | 0.86 | 9.0 | 3.69 | $MS^3$ | | 3 |
| 303.1227 | C17H20O5 | Dimer | -1.1 | 1.00 | 8.0 | 4.30 | | | 3 |
| 311.0915 | C18H16O5 | Dimer | -1.0 | 0.94 | 11.0 | 4.21 | | | 3 |
| 313.1434 | C19H22O4 | Dimer | -1.1 | 1.00 | 9.0 | 5.17 | | | 3 |
| | | | | 1.00 | | 5.74 | $MS^3$ | | 3 |
| 315.1225 | C18H20O5 | Dimer | -1.2 | 0.92 | 9.0 | 2.85 | | | 3 |
| | | | | 0.99 | | 2.93 | | | 3 |
| | | | | 1.04 | | 3.13 | | | 3 |
| | | | | 1.03 | | 3.45 | | | 3 |
| 317.1019 | C17H18O6 | Dimer | -1.1 | 0.92 | 9.0 | 4.49 | | | 3 |
| | | | | 1.01 | | 4.56 | | | 3 |
| | | | | 0.93 | | 4.63 | | | 3 |
| | | | | 0.94 | | 4.88 | $MS^3$ | | 2 |
| 317.1383 | C18H22O5 | Dimer | -1.1 | 1.00 | 8.0 | 3.54 | | | 3 |
| 319.1177 | C17H20O6 | Dimer | -1.0 | 0.85 | 8.0 | 5.00 | | | 3 |
| 329.1382 | C19H22O5 | Dimer | -1.2 | 1.00 | 9.0 | 2.99 | | | 3 |
| 331.1176 | C18H20O6 | Dimer | -1.1 | 1.00 | 9.0 | 3.50 | | | 3 |
| | | | | 0.95 | | 4.34 | | | 3 |

| | | | | | | | | | |
|---|---|---|---|---|---|---|---|---|---|
| | | | | 0.89 | | 4.41 | | | 3 |
| | | | | 0.93 | | 4.59 | | | 3 |
| | | | | 0.91 | | 4.69 | $MS^3$ | | 3 |
| 337.1071 | C20H18O5 | Dimer | -1.0 | 1.00 | 12.0 | 5.00 | | | 3 |
| 339.1227 | C20H20O5 | Dimer | -1.1 | 0.92 | 11.0 | 3.79 | | | 3 |
| 345.1331 | C19H22O6 | Dimer | -1.2 | 0.89 | 9.0 | 4.28 | | | 3 |
| | | | | 0.95 | | 4.46 | | | 3 |
| | | | | 0.97 | | 4.56 | | | 3 |
| | | | | 0.96 | | 4.60 | $MS^3$ | | 2 |
| | | | | 1.00 | | 6.86 | | | 3 |
| 355.1175 | C20H20O6 | Dimer | -1.2 | 0.93 | 11.0 | 4.63 | | | 3 |
| 357.1332 | C20H22O6 | Dimer | -1.1 | 0.91 | 10.0 | 1.20 | | | 3 |
| | | | | 0.97 | | 3.58 | | | 3 |
| | | | | 0.97 | | 4.60 | | | 3 |
| | | | | 0.86 | | 6.20 | | | 3 |
| 359.1491 | C20H24O6 | Dimer | -0.9 | 0.89 | 9.0 | 4.19 | | | 3 |
| | | | | 0.91 | | 5.45 | | | 3 |
| 365.1019 | C21H18O6 | Dimer | -1.1 | 0.97 | 13.0 | 5.48 | | | 3 |
| 373.1281 | C20H22O7 | Dimer | -1.2 | 0.92 | 10.0 | 5.45 | | | 3 |
| | | | | 0.91 | | 5.49 | | | 3 |
| 375.1437 | C20H24O7 | Dimer | -1.2 | 0.93 | 9.0 | 3.37 | | | 3 |
| | | | | 1.08 | | 4.66 | | | 3 |
| | | | | 0.94 | | 5.48 | | | 3 |
| | | | | 1.00 | | 5.57 | | | 3 |
| 387.1437 | C21H24O7 | Dimer | 1.1 | 1.03 | 14.0 | 5.14 | | | 3 |
| 397.1281 | C22H22O7 | Dimer | -1.2 | 0.89 | 12.0 | 5.24 | | | 3 |
| 405.1543 | C21H26O8 | Dimer | -1.2 | 0.86 | 9.0 | 4.78 | | | 3 |

| | | | | | | | | | |
|---|---|---|---|---|---|---|---|---|---|
| | | | | 0.89 | | 5.07 | | | 3 |
| 511.1963 | C28H32O9 | Trimer | -1.0 | 0.96 | 13.0 | 6.11 | | | |

**Table S4.** Identified phenolic compounds in the 2BDM190 sample including the detected [M-H]$^-$, determined chemical formulas, classified class, obtained mass difference, obtained $^{13}$C ratio, ring double bound (RDB) equivalent, retention times (RTs), obtained MS$^n$ spectra (*: MS$^n$ of identified fragment).

| [M-H]- | Chemical formula (neutral) | Classified class | Mass diff. (mDa) | $^{13}$C ratio | RDB | RTs (min) | MS$^n$ | Suggested compound | Identification confidence level |
|---|---|---|---|---|---|---|---|---|---|
| 121.0296 | C7H6O2 | Monomer | 0.1 | 1.03 | 5.0 | 2.50 | | 4-hydroxybenzaldehyde | 1 |
| 137.0245 | C7H6O3 | Monomer | 0.1 | 0.96 | 5.0 | 4.02 | | 4-hydroxybenzoic acid | 1 |
| 151.0400 | C8H8O3 | Monomer | 0.0 | 1.00 | 5.0 | 1.26 | MS$^3$ | Vanillin | 1 |
| 177.0556 | C10H10O3 | Monomer | -0.1 | 1.07 | 6.0 | 3.55 | | (E)-3,4-dihydroxy-benzylideneacetone | 3 |
| | | | | 0.89 | | 3.66 | | | 3 |
| 191.0712 | C11H12O3 | Monomer | -0.1 | 0.86 | 6.0 | 3.44 | | | 3 |
| | | | | 0.96 | | 3.61 | | | 3 |
| 195.0660 | C10H12O4 | Monomer | -0.3 | 0.88 | 5.0 | 1.64 | | Acetosyringone | 1 |
| | | | | 0.88 | | 3.23 | | 1-(3,4-dihydroxy-5-methoxyphenyl) propan-2-one | 3 |
| 227.0710 | C14H12O3 | Dimer | -0.3 | 1.06 | 9.0 | 3.55 | | | 3 |
| 243.0657 | C14H12O4 | Dimer | -0.6 | 0.93 | 9.0 | 3.41 | | | 3 |
| | | | | 1.07 | | 3.90 | | | 3 |
| 245.0813 | C14H14O4 | Dimer | -0.6 | 1.07 | 8.0 | 2.54 | | | 3 |
| | | | | 0.95 | | 4.04 | | | 3 |
| 255.0657 | C15H12O4 | Dimer | -0.6 | 0.88 | 10.0 | 4.43 | | | 3 |
| 257.0813 | C15H14O4 | Dimer | -0.6 | 1.00 | 9.0 | 2.86 | | | 3 |
| | | | | 0.89 | | 3.27 | | | 3 |
| | | | | 0.86 | | 3.34 | | | 3 |
| | | | | 1.05 | | 3.61 | | | 3 |
| | | | | 0.93 | | 3.82 | | | 3 |

| | | | | | | | | | |
|---|---|---|---|---|---|---|---|---|---|
| 259.0608 | C14H12O5 | Dimer | -0.4 | 1.03 | 9.0 | 3.61 | $MS^3$ | | 2 |
| 271.0970 | C16H16O4 | Dimer | -0.6 | 0.97 | 9.0 | 2.88 | | | 3 |
| | | | | 1.06 | | 3.63 | | | 3 |
| | | | | 0.85 | | 3.92 | | | 3 |
| | | | | 0.85 | | 4.04 | | | 3 |
| 273.0762 | C15H14O5 | Dimer | -0.6 | 0.93 | 9.0 | 2.52 | | | 3 |
| | | | | 1.07 | | 3.27 | $MS^3$ | | 3 |
| | | | | 1.02 | | 3.58 | $MS^3$ | | 3 |
| | | | | 1.03 | | 4.25 | | | 3 |
| 273.1127 | C16H18O4 | Dimer | -0.5 | 0.85 | 8.0 | 2.41 | | | 3 |
| 287.0918 | C16H16O5 | Dimer | -0.7 | 1.00 | 9.0 | 3.14 | | | 3 |
| 289.1078 | C16H18O5 | Dimer | -0.3 | 0.86 | 8.0 | 4.13 | | | 3 |
| | | | | 0.97 | | 4.21 | | | 3 |
| 297.0763 | C17H14O5 | Dimer | -0.5 | 1.00 | 11.0 | 4.24 | $MS^3$ | | 3 |
| 297.1491 | C19H22O3 | Dimer | -0.5 | 1.00 | 9.0 | 4.66 | | | 3 |
| 299.0920 | C17H16O5 | Dimer | -0.5 | 0.92 | 10.0 | 3.73 | | | 3 |
| 301.0711 | C16H14O6 | Dimer | -0.6 | 1.00 | 10.0 | 3.93 | $MS^3$ | | 2 |
| 301.1075 | C17H18O5 | Dimer | -0.6 | 0.93 | 9.0 | 2.86 | | | 3 |
| | | | | 1.06 | | 3.08 | | | 3 |
| | | | | 0.87 | | 3.29 | | | 3 |
| 303.0868 | C16H16O6 | Dimer | -0.6 | 0.87 | 9.0 | 2.71 | $MS^3$ | | 3 |
| 311.0919 | C18H16O5 | Dimer | -0.6 | 0.95 | 11.0 | 4.21 | | | 3 |
| 313.1438 | C19H22O4 | Dimer | -0.7 | 1.00 | 9.0 | 5.18 | | | 3 |
| | | | | 1.10 | | 5.75 | $MS^3$ | | 3 |
| 315.0867 | C17H16O6 | Dimer | -0.7 | 0.85 | 10.0 | 1.26 | | | 3 |
| | | | | 0.87 | | 3.57 | | | 3 |
| | | | | 0.88 | | 3.76 | | | 3 |

| | | | | | | | | | |
|---|---|---|---|---|---|---|---|---|---|
| | | | | 0.88 | | 3.99 | | | 3 |
| 315.1231 | C18H20O5 | Dimer | -0.7 | 1.06 | 9.0 | 2.94 | $MS^3$ | | 2 |
| | | | | 1.08 | | 3.12 | $MS^3$ | | 2 |
| | | | | 1.00 | | 3.44 | | | 3 |
| 323.0919 | C19H16O5 | Dimer | -0.6 | 0.91 | 12.0 | 3.86 | | | 3 |
| | | | | 0.90 | | 4.75 | | | 3 |
| 329.1024 | C18H18O6 | Dimer | -0.6 | 1.02 | 10.0 | 3.79 | $MS^3$ | | 2 |
| | | | | 1.00 | | 4.45 | | | 3 |
| 329.1387 | C19H22O5 | Dimer | -0.7 | 0.92 | 9.0 | 6.18 | | | 3 |
| 331.0815 | C17H16O7 | Dimer | -0.8 | 0.93 | 10.0 | 3.92 | | | 3 |
| 331.1180 | C18H20O6 | Dimer | -0.7 | 1.00 | 9.0 | 4.28 | | | 3 |
| 333.0972 | C17H18O7 | Dimer | -0.8 | 0.91 | 9.0 | 3.78 | | | 3 |
| 339.1231 | C20H20O5 | Dimer | -0.7 | 0.89 | 11.0 | 3.78 | | | 3 |
| 343.1181 | C19H20O6 | Dimer | -0.6 | 0.96 | 10.0 | 2.77 | | | 3 |
| | | | | 1.07 | | 3.60 | | | 3 |
| | | | | 1.00 | | 3.72 | | | 3 |
| | | | | 0.85 | | 4.89 | | | 3 |
| 343.1543 | C20H24O5 | Dimer | -0.8 | 1.00 | 9.0 | 3.01 | | | 3 |
| | | | | 0.94 | | 3.11 | | | 3 |
| 345.0971 | C18H18O7 | Dimer | -0.9 | 0.93 | 10.0 | 3.78 | | | 3 |
| 345.1337 | C19H22O6 | Dimer | -0.6 | 1.02 | 9.0 | 4.62 | $MS^3$ | | 2 |
| | | | | 0.94 | | 6.87 | | | 3 |
| 349.1073 | C21H18O5 | Dimer | -0.8 | 1.00 | 13.0 | 3.95 | | | 3 |
| 357.1336 | C20H22O6 | Dimer | -0.7 | 0.91 | 10.0 | 2.66 | | | 3 |
| | | | | 0.90 | | 3.57 | | | 3 |
| | | | | 0.96 | | 4.22 | | | 3 |
| 361.0920 | C18H18O8 | Dimer | -0.9 | 0.96 | 10.0 | 3.90 | | | 3 |

| | | | | | | | | | |
|---|---|---|---|---|---|---|---|---|---|
| 361.1647 | C20H26O6 | Dimer | -0.9 | 1.06 | 8.0 | 5.06 | $MS^3$ | | 2 |
| | | | | 1.00 | | 5.31 | | | 3 |
| 365.1024 | C21H18O6 | Dimer | -0.6 | 0.92 | 13.0 | 5.48 | | | 3 |
| 373.1285 | C20H22O7 | Dimer | -0.8 | 0.99 | 10.0 | 5.45 | | | 3 |
| | | | | 0.92 | | 5.51 | | | 3 |
| 375.1442 | C20H24O7 | Dimer | -0.7 | 0.95 | 9.0 | 4.66 | | | 3 |
| 379.1179 | C22H20O6 | Dimer | -0.8 | 0.99 | 13.0 | 4.13 | | | 3 |
| 387.1439 | C21H24O7 | Dimer | -1.0 | 1.02 | 10.0 | 4.36 | | | 3 |
| 389.1595 | C21H26O7 | Dimer | -1.1 | 1.00 | 9.0 | 4.71 | | | 3 |
| | | | | 0.92 | | 5.16 | | | 3 |
| 403.1390 | C21H24O8 | Dimer | -0.8 | 1.04 | 10.0 | 3.93 | | | 3 |
| 403.1757 | C22H28O7 | Dimer | -0.5 | 1.03 | 9.0 | 4.54 | | | 3 |
| 405.1546 | C21H26O8 | Dimer | -0.9 | 0.87 | 9.0 | 4.75 | | | 3 |
| | | | | 0.85 | | 5.07 | | | 3 |
| 409.1283 | C23H22O7 | Dimer | -1.0 | 1.00 | 13.0 | 4.19 | | | 3 |
| | | | | 0.94 | | 4.84 | | | 3 |
| 423.1076 | C23H20O8 | Dimer | -0.9 | 0.99 | 14.0 | 5.12 | | | 3 |
| 433.1495 | C22H26O9 | Dimer | -0.6 | 1.04 | 10.0 | 4.36 | | | 3 |
| | | | | 0.88 | | 4.42 | | | 3 |
| 451.1759 | C26H28O7 | Trimer | -0.3 | 0.86 | 13.0 | 4.62 | | | 3 |
| 465.1910 | C27H30O7 | Trimer | -0.9 | 1.01 | 13.0 | 4.42 | | | 3 |
| 483.2019 | C27H32O8 | Trimer | -0.5 | 0.96 | 12.0 | 5.19 | | | 3 |
| | | | | 0.85 | | 5.40 | | | 3 |
| | | | | 0.95 | | 5.83 | | | 3 |
| 511.1965 | C28H32O9 | Trimer | -0.8 | 0.93 | 13.0 | 5.56 | | | 3 |
| | | | | 0.90 | | 6.12 | | | 3 |
| | | | | 0.92 | | 6.16 | | | 3 |

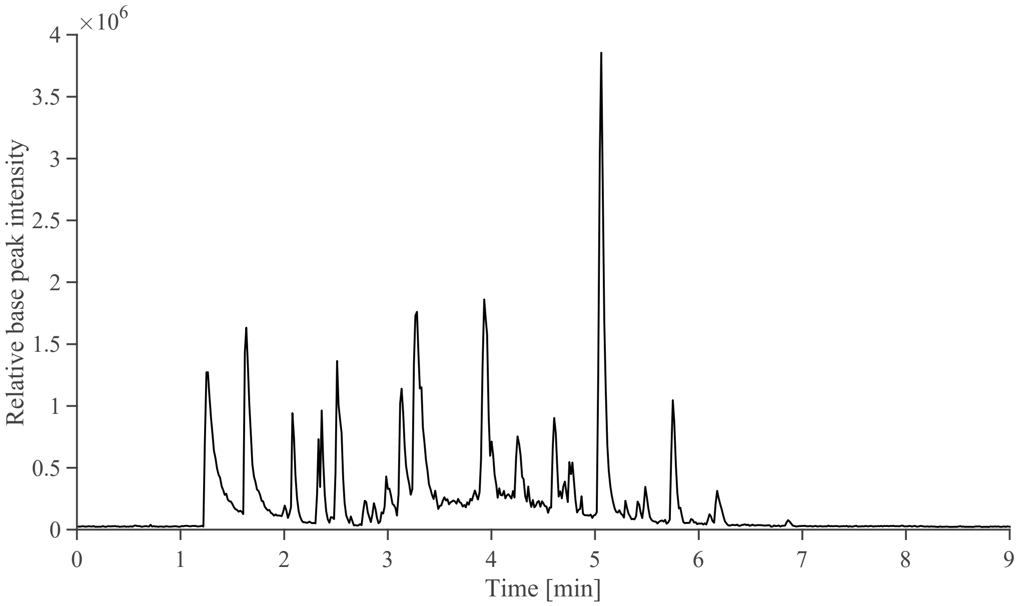

Figure S1. BPI of BDM170 (data already presented previously in  Prothmann et al. 2020 [1].

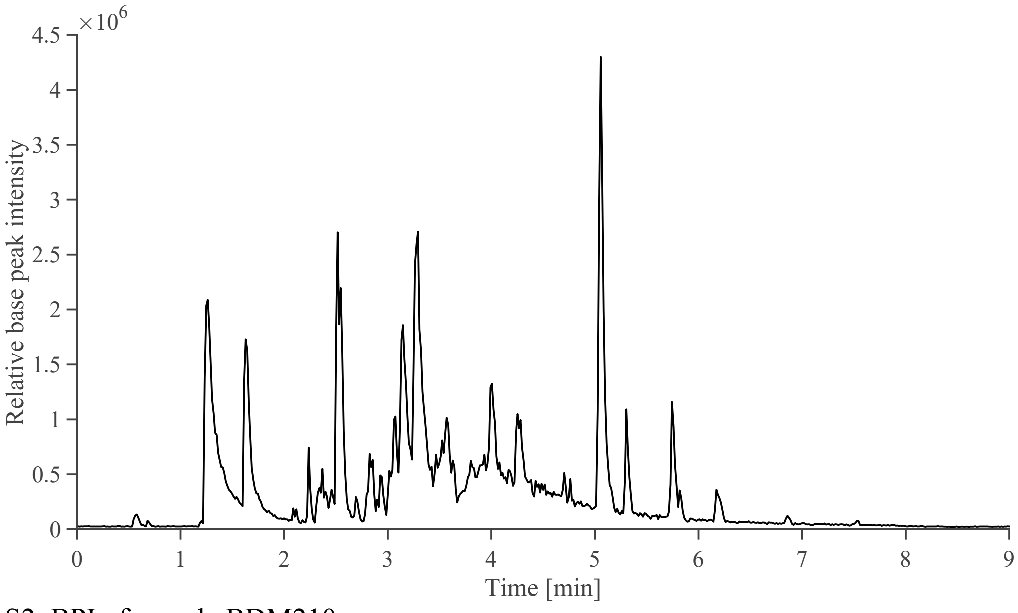

Figure S2. BPI of sample BDM210.

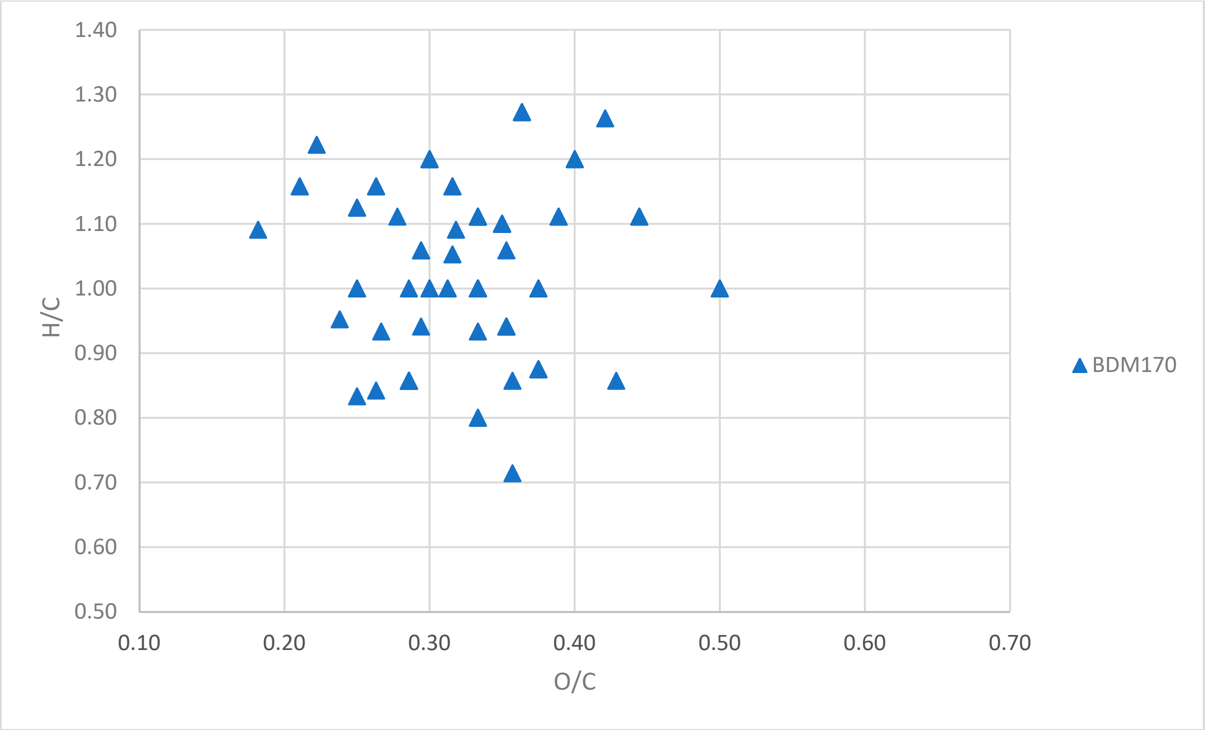


Figure S3. Van-Krevelen plot of sample BDM170.

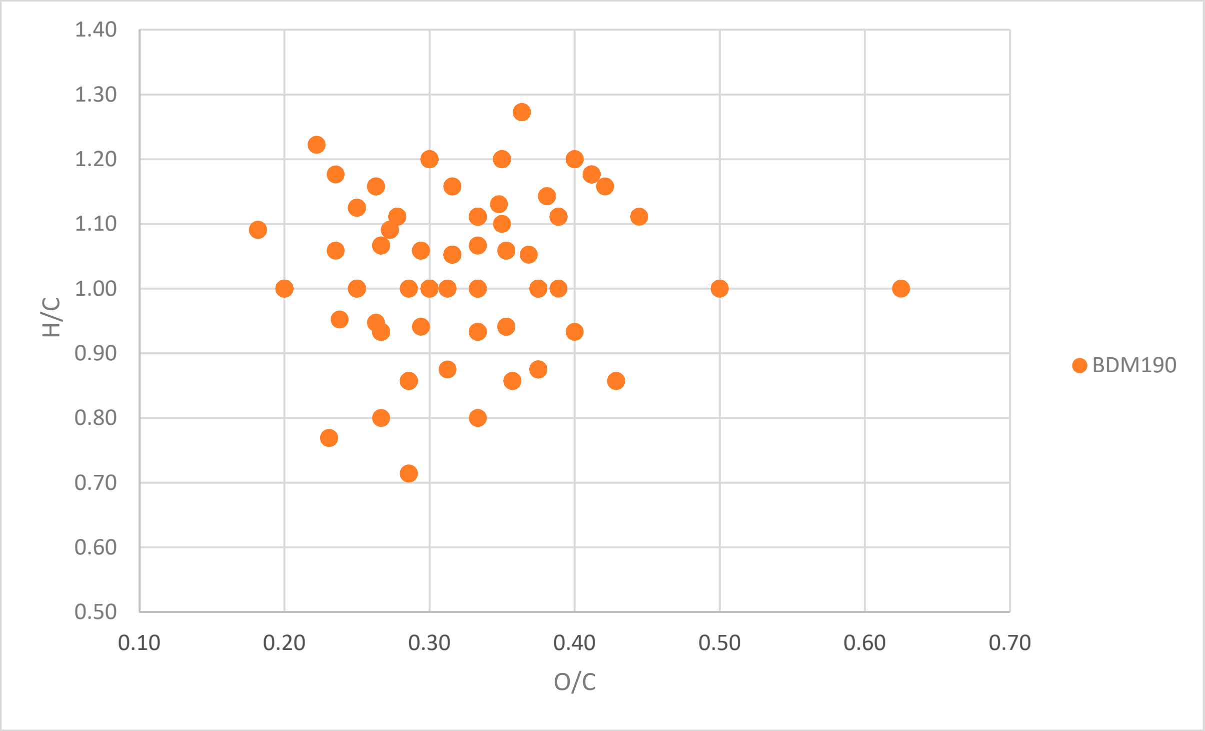


Figure S4. Van-Krevelen plot of sample BDM190.

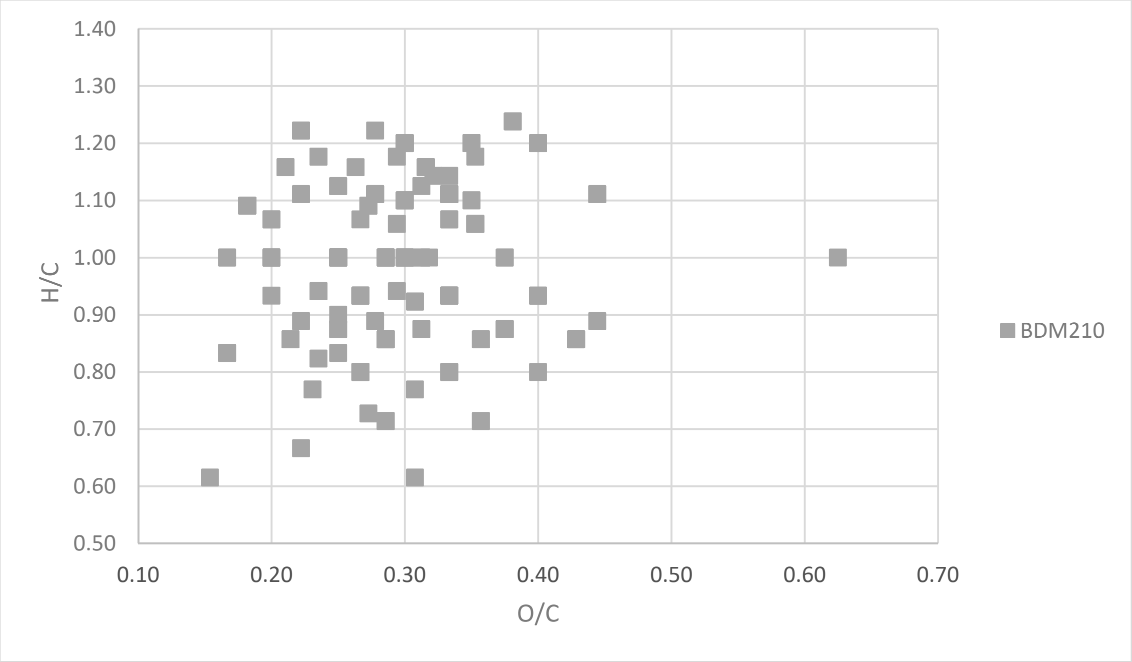

Figure S5. Van-Krevelen plot of sample BDM210.

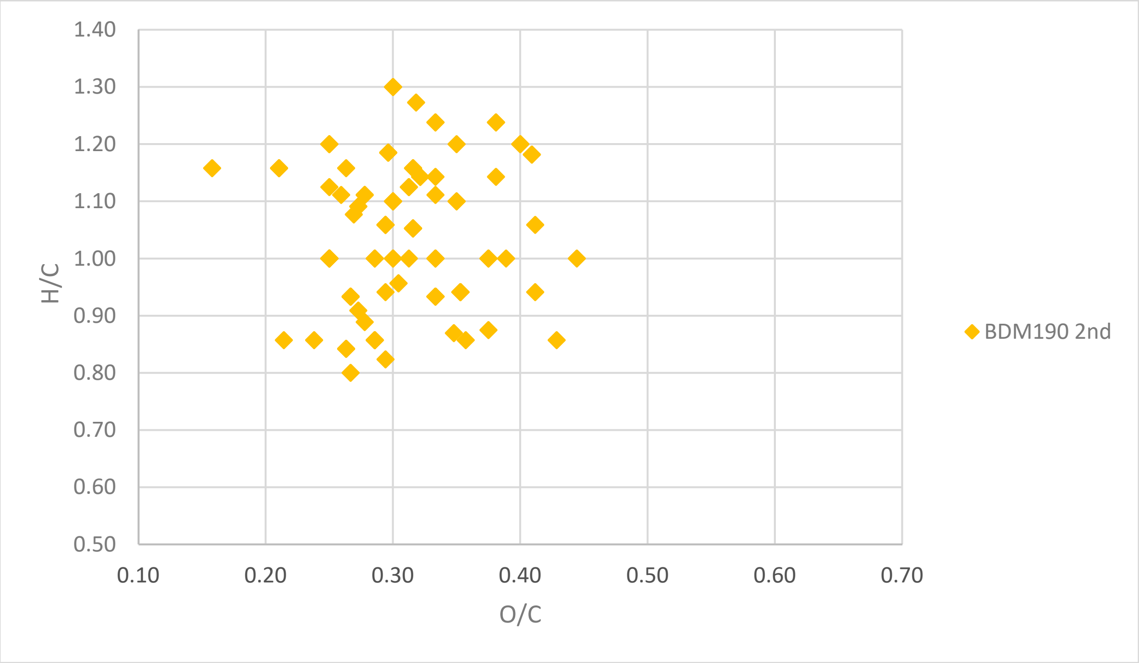

Figure S6. Van-Krevelen plot of sample 2BDM190.

## Reference


[1] J. Prothmann, K. Li, C. Hulteberg, P. Spégel, M. Sandahl, C. Turner, Nontargeted Analysis Strategy for the Identification of Phenolic Compounds in Complex Technical Lignin Samples, ChemSusChem. (2020) 4605–4612. https://doi.org/10.1002/cssc.202000951.
